# Simulated intention-to-treat analysis based on clinical parameters of patients at high risk for sleep apnea derivated to respiratory polygraphy

**DOI:** 10.5935/1984-0063.20180030

**Published:** 2018

**Authors:** Eduardo Enrique Borsini, Magali Blanco, Glenda Ernst, Paulina Montenegro, Alejandro Salvado, Carlos Nigro

**Affiliations:** 1 Hospital Británico de Buenos Aires, Sleep Units - Buenos Aires City - Buenos Aires - Argentina.; 2 Hospital Británico de Buenos Aires, Center for Respiratory Medicine - Buenos Aires City - Buenos Aires - Argentina.; 3 Hospital Alemán, Sleep Units - Buenos Aires City - Buenos Aires - Argentina.

**Keywords:** Sleep Apnea Syndromes, Continuous Positive Airway Pressure, Decision Making

## Abstract

**Purpose:**

Obstructive Sleep Apnea-Hypopnea Syndrome (OSAS) is a public health problem. We designed a pilot study to validate empiric indication of CPAP therapy in a population with moderate-to-high pre-test probabilities who underwent self-administered home-based respiratory polygraphy (RP).

**Methods:**

A cross-sectional simulation study was performed. CPAP therapy could be indicated by two independent blind observers. Observer 1´s decision was based on the results of STOP-BANG (SBQ) and Epworth Sleepiness Scale (ESS) and Observer 2 used all objective data provided by RP + SBQ + ESS.

**Results:**

We evaluated 1763 patients; 1060 men and 703 women (39.2%) with a mean age of 53.6±13.8 and a body mass index (BMI) of 32.8±7.5 kg/m2. We found evidence of mild (34.1%), moderate (26.6%), and severe (18.3%) There were Apnea-Hypopnea Index (AHI) relationship between > 5 or < 5 SBQ and RP AHI (*p*<0.05). BMI > 25 kg/m^2^ + snoring (S) + observed apnea (O) + 1 of the following: ESS > 11, hypertension (HT) or > 5 SBQ components showed sensitivity of 40% (CI95%: 37.3-43) and specificity of 95.1% (CI93.4-96.4). The performance of 5 SBQ components with regard to gender and empirical CPAP therapy was; (women vs. men): AUC-ROC 0.625 (CI95%: 0.599-0.651) *vs*. 0.70 (CI95%: 0.68-0.72), *p*<0.01, respectively.

**Conclusions:**

STOP-BANG and ESS made it possible to indicate CPAP reliably (low rate of false-positive results) in 20-40% of patients who needed such therapy according to clinical history and RP results. These clinical criteria performed better in male.

## INTRODUCTION

Obstructive Sleep Apnea-Hypopnea (OSA) is a public health problem due to its high prevalence and morbimortality^1^.

It has been described, the estimated prevalence of sleep-disordered breathing, defined as an apnea-hypopnea index > 5 events per hour, was 9 percent for women and 24 percent for men, while 2 percent of women and 4 percent of men gathered minimal diagnostic criteria for the sleep apnea syndrome (apnea-hypopnea index > 5 and daytime excessive somnolence)^2^^,^^3^. According to recent data, the prevalence of OSA in the general population of Latin America is 32%^4^.

In general, OSA diagnosis is confirmed through polysomnography (PSG), though duly validated respiratory polygraphy (RP) is also accepted in populations with a high clinical probability of suffering from OSA^5^^,^^6^. Considering the OSA - related risk of car accidents^7^ and cardiovascular morbidity^8^, and the documented effectiveness of continuous positive airway pressure (CPAP) therapy, the study of clinical variables for OSA diagnosis should be a priority. Some authors have proposed different alternatives, such as assessing clinical^9^^,^^10^, functional^11^, or anthropometric^12^ parameters to detect severe OSA or calculate its AHI.

Though several studies have assessed the use of diagnostic tools based on predictive equations^9^^,^^13^^,^^14^, it is difficult to make comparisons or extrapolate results because of the different combinations of variables and the heterogeneity of study populations. In general, these predictive equations have presented high sensitivity (78-95%) and low specificity (41-63%) for different AHI cutoff points (generally, between 5 and 20) in populations with a different prevalence of OSA^9^^,^^10^.

A questionnaire to indicate CPAP (QPCPAP) in patients with suspected OSA^15^ has recently been validated. QPCPAP is based on the criteria of the Berlin questionnaire, Epworth Sleepiness Scale (ESS), and a general health status questionnaire. QPCPAP results were compared against a combination of full polysomnography findings + SEPAR (acronym in spanish for Spanish Society of Respiratory Pathology and Thoracic Surgery) or AAMS (American Academy of Sleep Medicine) criteria^4^^,^^15^^-^^17^. Authors concluded that QPCPAP allowed them to make a reliable indication of CPAP in approximately 30% of clinical patients (97-98% specificity, positive likelihood ratio > 10).

There is limited information about the usefulness of clinical criteria based on STOP-BANG QUESTIONNAIRE (SBQ) to indicate empirical CPAP therapy for subjects with suspected OSA. Thus, we designed a pilot study to validate SBQ as a tool to guide the indication of CPAP in clinical populations with moderate-high OSA according to self-administered home-based respiratory polygraphy (RP).

## MATERIALS AND METHODS

We checked the data systematically collected in the database of the Sleep Laboratory of Hospital Británico de Buenos Aires between January 2012 and December 2016.

### Population and design study

**Figure 1:**
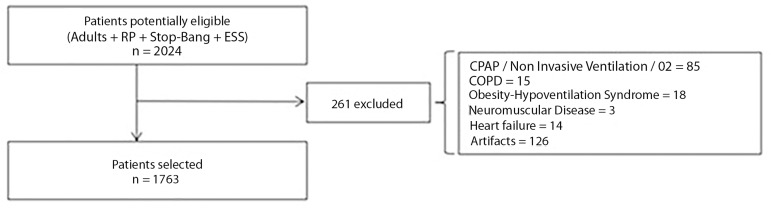
Flow chart for patients selection.

This was a cross-sectional simulated intention-to-treat analysis. We selected from a database 1763 adult patients with complete anthropometric data (BMI, neck circumference), SBQ^18^ and Epworth Sleepiness Scale (ESS)^19^ who were subjected to RP with a minimum valid total recording time (TRT) of 4 hours. Patients on oxygen or CPAP/non-invasive ventilation, with COPD diagnosis, obesity-hypoventilation syndrome, heart failure, neuromuscular disease or respiratory polygraphy with artifacts in airflow, respiratory effort or SO_2_, were excluded (Figure 1). The information was recorded on an Excel worksheet. Precautions were taken to delete data that could make it possible to identify patients or breach data confidentiality during their processing. The protocol was approved by the Institutional Ethics and Review Committee pursuant to the Declaration of Helsinki.

### Respiratory Polygraphy

Portable Apnea Link Air device (ResMed. Sydney. Australia) with nasal pressure cannula, thoracic effort sensor, and oximetry were used for self-administered home-based RP. Recordings were taken at night and later edited manually by pulmonologists trained in the standards and guidelines of the American Academy of Sleep Medicine (AASM)^6^^,^^20^. Apnea was defined as a decrease in airflow by > 80% of baseline for ≥ 10 seconds and hypopnea as a 50% drop for ≥ 10 seconds associated with ≥ 3% oxygen desaturation. AHI was calculated as the number of apnea/hypopnea events per hour (events/hour) of valid TRT. Patients were classified as non-OSA (AHI < 5), mild-OSA (AHI between ≥ 5 and < 15), moderate-OSA (AHI between ≥ 15 and < 30), and severe-OSA (AHI ≥ 30) patients.

### Empiric decision to treat with CPAP

We simulated a situation in which two blind, independent observers could indicate CPAP therapy. Both observers are experts in sleep medicine responsible for respective sleep units. Observer 1 based the indication on SBQ and ESS results (test); while Observer 2 based the indication on objective data from RP + SBQ + ESS (reference method).

The clinical criteria used by Observer 1 to indicate CPAP to patients with suspected OSA were based on previously published data^15^. Thus, patients with overweight or obesity (BMI > 25 kg/m^2^), severe snoring, and observed apnea with excessive daytime sleepiness (ESS > 11) or hypertension (HT) with > 5 SBQ components in any combination could become candidates for CPAP therapy.

Observer 2 based the indication of CPAP on AHI and significant daytime sleepiness (ESS > 11) or hypertension according to the guidelines of the Spanish Society of Pneumonology and Thoracic Surgery (SEPAR, for its Spanish acronym)^5^^,^^16^. Table 1 shows the criteria used by each observer.

**Table 1:** Criteria to indicate CPAP.

Observer 1 (Test)
*A Criteria*
BMI > 25 kg/m^2^ + loud snoring (S) + observed apnea (O) + daytime sleepiness (Epworth > 11)
*B Criteria*
BMI > 25 kg/m^2^ + loud snoring (S) + observed apnea (O) + daytime sleepiness (Epworth > 11) or hypertension
HTN and > 5 components SBQ in any combination
**Observer 2 (Reference method)**
1. Apnea-Hypopnea Index (AHI) ≥ 30
2. AHI ≥ 5 and < 30 + one of the following symptoms:
• Daytime sleepiness (Epworth > 11)
• Hypertension
• > 5 components SBQ in any combination

BMI: body mass index (kg/m^2^); CPAP: continuous positive airway pressure

### Statistical analysis

Distribution of variables was assessed using Kolmogorov-Smirnov frequency histogram. Results were presented as percentages for categorical variables and as mean or median and standard deviation or interquartile range (IQR_25-75%_) for numerical variables. To analysis differences between categorical variables Fisher test was used. The area under the ROC curve (AUC-ROC test) was analyzed and sensitivity, specificity and positive and negative likelihood ratio were calculated. The following software were used: Prism 7 (Graph Pad, La Jolla, CA) and MedCalc Statistical Software version 17.9 (MedCalc Software, Ostend, Belgium; http://www.medcalc.org; 2017).

## RESULTS

**Figure 2:**
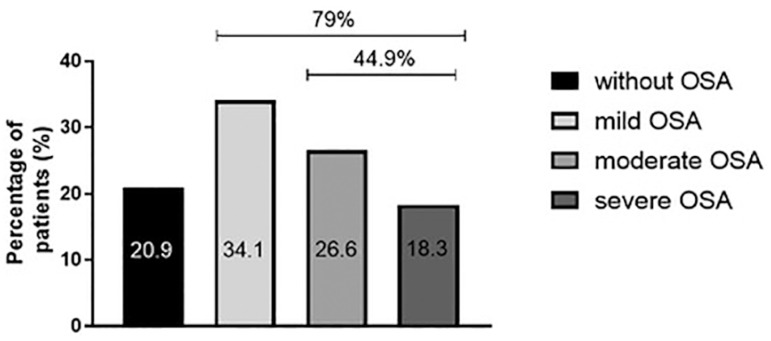
Distribution by group is severity according to AHI.

We evaluated 1763 patients (703 women-39.9%) with a median age, BMI and AHI of; 53.6 (±13.8) years, 32.8 (±7.5) kg/m^2^ and 18.7 (±17.1) events / hour, respectively. 79% of the population had a diagnosis of OSA. According to AHI; 34.1% patients had mild OSA, 26.6% had moderate OSA, and 18.3% had severe OSA (Figure 2). The time of oxygen saturation below 90% was 18.4±26.7% of TRT. Table 2 shows the characteristics of study population.

**Table 2:** Study population characteristics.

Parameters n=1763	
Sex n (%)	1060 men (60.1), 703 women (39.9)
Age (years)*	53.6 (44 - 64)
BMI (kg/m^2^)*	32.8 (27.4 37.3 )
Epworth*	7 (4 - 11)
Epworth > 11 n (%)	429 (24.3)
Tiredness n (%)	1241 (70.4)
Hypertension n (%)	818 (46.4)
Respiratory polygraphy	
- Total recording time (min.)*	425 (373 - 485)
- Evaluation period (min.)*	400 (342 - 454)
- AHI*	18.7 (9.2 - 24.9)
- ODI3*	16 (8 - 28)
OSA n (%)	1394 (79)
- AHI ≥ 5 - < 15 n (%)	601 (34.1)
- AHI ≥ 15 - < 30 n (%)	470 (26.6)
- AHI ≥ 30 n (%)	323 (18.3)

* Values expressed as median and percentiles 25-75%.

AHI: apnea/hypopnea index. ODI3: oxygen desaturation Index of 3%. OSA: obstructive sleep apnea.

Women had a lower AHI and a higher BMI than men (AHI: 9.9, IQR_25-75%_ 4.8 - 18 *vs.* 19.2, IQR_25-75%_ 8.2 - 30.1, *p*<0.01; BMI: 33, IQR_25-75%_ 27.5 - 40.2 *vs.* 30.8, IQR_25-75%_ 27.4 - 35.1, < 0.01).

**Figure 3:**
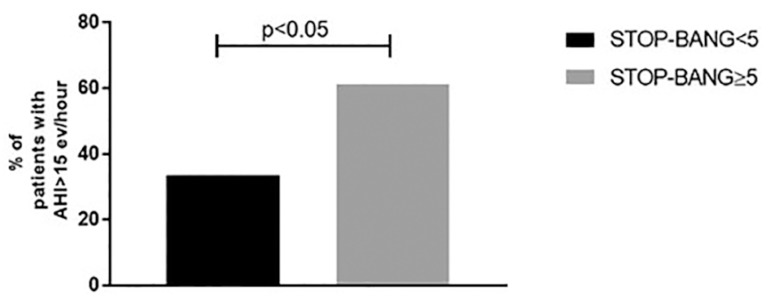
Relationship between > 5 or < 5 SBQ components and AHI.

Five percent of cases (89 cases) presented complete SBQ (8 components) and there was a statistically significant relationship (*p*<0.05) between ≥ 5 or < 5 SBQ and RP AHI (Figure 3). Table 3 shows the distribution of SBQ variables.

**Table 3:** Components and combinations found in STOP-BANG questionnaire.

Variables	Frequency	Percentage
**S** (Snoring)	1120	63.5
**T** (Tiredness)	1241	70.4
**O** (Observed Apneas)	895	50.8
**P** Hypertension known or previously diagnosed	815	46.2
**B** BMI (body mass index) > 35 kg/m^2^	713	40.4
**A** Age > 50 years old	1132	64.2
**N** (neck circumference) > 40 cm	1060	59.43
**G** Gender (Male)	1052	60.1
**STOP-BANG** (8 components)	89	5.04
**STOP**	304	17.2
**BANG**	221	12.5
**STOP-BANG** (≥ 5 components)	898	78.06
**STOP-BANG** (< 5 components)	865	21.93

The area under the ROC curve (AUC-ROC), sensitivity and specificity of criteria A and B are shown in Table 4. As it can be seen, criteria A showed a lower sensitivity but a higher specificity and positive likelihood ratio than criteria B. There were 39 (criteria B) false positive cases. As compared to true negative patients, they had a lower AHI (3.1 *vs.* 6.4, *p* 0.02), a higher BMI (32.3 kg/m^2^ vs. 29.3 kg/m^2^, *p*<0.01) and a greater prevalence of daytime sleepiness and hypertension (Epworth > 11: 8.5% *vs.* 38.4%, hypertension: 12.6% *vs.* 77%, *p*<0.01). Criteria A and B performed better in men than in women (Table 5).

**Table 4:** Accuracy of clinical parameters to indicate CPAP.

Clinical criteria to indicate CPAP	Sensitivity (%) (CI95%)	Specificity (%)(CI95%)	PLR (CI95%)	NLR (CI95%)	AUC-ROC (SE)
A criteria	20.7 (18 - 23)	98.1 (97 - 99)	10.6 (6.3 - 17.8)	0.81 (0.8 - 0.8)	0.594 (0.0069)
B criteria	40.9 (38 - 44)	94.9 (93 - 96)	8.1 (5.9 - 11.1)	0.62 (0.59 - 0.62)	0.679 (0.0087)

P/NLR: positive and negative likelihood ratio; CPAP: continuous positive airway pressure; CI95%: confidence interval 95%.

**Table 5:** Accuracy of clinical parameters to indicate CPAP therapy in women and men.

Clinical criteria to indicate CPAP	Sensitivity (%) (CI95%)	Specificity (%) (CI95%)	PLR (CI95%)	NLR (CI95%)	AUC-ROC (SE)
**Women**					
A criteria	13.2 (10 - 17)	98.4 (96.5 - 99.4)	8.2 (3.5 - 19)	0.88 (0.8 - 0.9)	0.56 (0.0099)
B criteria	30.4 (25.5 - 36)	95.1 (92.4 - 97)	6.3 (3.9 - 10)	0.73 (0.7 - 0.8)	0.63 (0.0138)
**Men**					
A criteria	24.3 (21 - 28)	97.8 (96 - 99)	10.9 (5.6 - 21.1)	0.77 (0.7 - 0.8)	0.611 (0.0092)
B criteria	46.3 (42.3 - 50)	94.8 (92.1 - 96.7)	8.8 (5.8 - 13.5)	0.57 (0.5 - 0.6)	0.704 (0.0112)

P/NLR: positive and negative likelihood ratio. CI95%: confidence interval 95%. AUC-ROC (SE): area under curve (standard error); CPAP: continuous positive airway pressure. *p<0.01

The performance of 5 SBQ components with regard to gender and empirical CPAP therapy was; (women *vs.* men): AUC-ROC 0.625 (CI95%: 0.599-0.651) *vs.* 0.70 (CI95%: 0.68-0.72), *p*<0.01, respectively.

## DISCUSSION

The main finding of this simulation study conducted in a cohort with high prevalence of sleep apnea suggests that it is possible to indicate empirical CPAP therapy based only on clinical criteria for approximately one third of the population with RP-confirmed OSA. However our results showed that clinical criteria are weak (low sensitivity).

In 2008, the STOP (*Snore, Tired, Observed apnea, and Pressure*) questionnaire was validated. It consists of four yes/no questions and it was designed by Canadian researchers to track OSA in surgical populations^18^. In the original publication, the STOP questionnaire showed a variable predictive value for each AHI cut-off point of supervised PSG (AUC-ROC 0.73 for AHI > 5 events/hour and 0.76 for AHI > 30 events/hour).

The addition of anthropometric parameters; (BANG questionnaire)- *Body Mass Index (BMI >35 kg/m*^2^ *), Age (>50 years of age), Neck (neck circumference > 40 cm), and Gender (being male)* -increased the sensitivity and positive predictive value of the scale (AUC-ROC 0.80 for AHI >5 events/hour and 0.82 for >30 events/hour) and allowed physicians to identify patients at high risk for sleep apnea.

At present, SBQ is used in NON-surgical populations and has been validated as a clinical tool to identify patients with OSA in centers that use RP^21^. Trenaman et al.^22^ developed a web-based model of a patient decision aid which focuses on two first-line treatment options; CPAP and mandibular advancement including SBQ, and indicated acceptable performance in this model for participants. Although the study is interesting since it exposes a model of shared decisions, its sample was limited and the analysis focused on the performance of a prototype. Our strategy based on the use of clinical data gathered during routine visits and SBQ yielded AUC-ROC values between 0.6 and 0.7 with high specificity (95 to 98%) and a positive likelihood ratio of about 10, which makes this tool a reliable test to start treatment with CPAP (low false positive rate).

One of the limitations of our approach is that two thirds of OSA patients with a potential need for CPAP therapy were not identified through clinical criteria (false-negative results) since they did not report frequent apneas or reported less self-perceived daytime symptoms. This may be partly due to the fact that questionnaires were filled out by patients themselves without the collaboration of those who live with them.

On the other hand, the specificity of > 5 SBQ was high (> 90%), which offers the advantage of preventing the implementation of CPAP therapy in patients without definitive indication. Observer 1 unnecessarily prescribed CPAP therapy 2 to 5% of patients (false-positive results) using criteria A or B as clinical approach. A wrong and unnecessary indication of empirical CPAP could result in low adherence or minor adverse events without risk to the patient. These intolerances may also occur in patients with a correct indication of CPAP therapy after RP.

The availability of a tool for simple clinical data collection offers several advantages. Firstly, patients with daytime sleepiness or cardio-metabolic comorbidities and at risk for vascular events or accidents could start treatment early, even at the primary level of care or during evaluation of intercurrent events (i.e. admissions to hospital due to cardiovascular episodes, stroke, or accidents caused by excessive sleepiness). Secondly, such tool could be useful in the case of long waiting lists for sleep tests. Finally, it could also lower costs, since almost one third of patients would not require a sleep test for initial diagnosis.

Anttalainen et al.^23^ developed a model whereby two experts used detailed clinical records and supplementary tests to initiate empirical CPAP therapy. Both obtained an acceptable level of agreement for 52% of the patients but with a high rate of false-positive results (11-26%). Nigro et al.^15^ findings are similar to ours. They used a similar strategy (PSG) based on clinical data from the Berlin questionnaire to show that in one third of cases it is possible to indicate empirical CPAP based on patients’ context.

This strategy to prescribe CPAP based on the SBQ and the Epworth Sleepiness Scale, performed better in men than in women (Table 5). This may be related to the fact that women report apnea and loud snoring less frequently than men^24^^,^^25^. In line with these publications, we observed that women reported less snoring and apneas than men (S: 59% *vs.* 67%, *p*<0.05, OR: 38% *vs.* 59%, *p*<0.001).

### Limitations

Our study has several limitations. Our study was based on cross-sectional model what entails the typical limitations of this type of design. It is also necessary to point out some methodological limitations. The first one resides in the fact that we do not have all cardiovascular risk data since SBQ only asks about known history of HT and this could result in fewer indications of CPAP therapy. On the other hand, there is no consensus about which OSA patients should receive CPAP therapy or the role of daytime symptoms.

The American Academy of Sleep Medicine recommends CPAP in patients with a respiratory disturbance index (RDI) ≥ 15 events/hour or between ≥ 5 and < 15 + sleepiness. SEPAR guidelines, however, recommend treatment with CPAP in patients with RDI ≥ 30 events/hour or between 5 and 30 associated with daytime sleepiness (ESS > 11) or comorbidities. Like in other areas of medicine, the decision to treat OSA patients with CPAP lies with the treating physician, which may result in variations in observers and sensitivity / specificity results. Even though we have included daytime tiredness among the symptoms that may result in an indication of CPAP therapy based on ESS (> 11) or frequent tiredness (*T=Tired*), this symptom is not explicitly included in current recommendations. Inclusion of this symptom in theoretical models increased the number of subjects who required CPAP by approximately 30%.

Lastly, to assess the accuracy of this strategy in real life, it would be necessary to conduct a prospective multicenter study based on the same pattern.

## CONCLUSIONS

According to our data, STOP-BANG and ESS could be useful to indicate CPAP reliably (low rate of false-positive results) in 20-40% of patients in need for CPAP according to their clinical history and RP results. These clinical criteria performed better in male patients and were similar to previous studies using PSG.
